# CD40-signalling abrogates induction of RORγt^+^ Treg cells by intestinal CD103^+^ DCs and causes fatal colitis

**DOI:** 10.1038/ncomms14715

**Published:** 2017-03-09

**Authors:** Christian Barthels, Ana Ogrinc, Verena Steyer, Stefanie Meier, Ferdinand Simon, Maria Wimmer, Andreas Blutke, Tobias Straub, Ursula Zimber-Strobl, Esther Lutgens, Peggy Marconi, Caspar Ohnmacht, Debora Garzetti, Bärbel Stecher, Thomas Brocker

**Affiliations:** 1 Institute for Immunology, LMU Munich, Großhaderner Strasse 9, Planegg-Martinsried 82152, Germany; 2 Center of Allergy Environment (ZAUM), Helmholtz Center and TU Munich, Neuherberg 85764, Germany; 3 Section of Animal Pathology, Department of Veterinary Clinical Sciences, LMU Munich, Munich 80539, Germany; 4 Bioinformatics core unit, BMC, LMU Munich, Großhaderner Strasse 9, Planegg-Munich 82152, Germany; 5 Helmholtz Zentrum München, Research Unit Gene Vectors, Munich 81377, Germany; 6 Institut für Prophylaxe und Epidemiologie der Kreislaufkrankheiten, LMU Munich, Munich 80336, Germany; 7 Department of Medical Biochemistry, AMC, Amsterdam 1105AZ, The Netherlands; 8 Department of Life Sciences and Biotechnology, University of Ferrara, Ferrara 44121, Italy; 9 Max von Pettenkofer Institute of Hygiene and Medical Microbiology, German Center for Infection Research (DZIF), Partner Site Munich, LMU Munich, Munich 80336, Germany

## Abstract

Immune homeostasis in intestinal tissues depends on the generation of regulatory T (Treg) cells. CD103^+^ dendritic cells (DCs) acquire microbiota-derived material from the gut lumen for transport to draining lymph nodes and generation of receptor-related orphan γt^+^ (RORγt^+^) Helios^−^-induced Treg (iTreg) cells. Here we show CD40-signalling as a microbe-independent signal that can induce migration of CD103^+^ DCs from the lamina propria (LP) to the mesenteric lymph nodes. Transgenic mice with constitutive CD11c-specific CD40-signalling have reduced numbers of CD103^+^ DCs in LP and a low frequency of RORγt^+^Helios^−^ iTreg cells, exacerbated inflammatory Th1/Th17 responses, high titres of microbiota-specific immunoglobulins, dysbiosis and fatal colitis, but no pathology is detected in other tissues. Our data demonstrate a CD40-dependent mechanism capable of abrogating iTreg cell induction by DCs, and suggest that the CD40L/CD40-signalling axis might be able to intervene in the generation of new iTreg cells in order to counter-regulate immune suppression to enhance immunity.

## Abstract (web summary)

CD103^+^ dendritic cells induce iTreg cells to maintain immune balance in the gut, but how CD40-signalling regulates this process is unclear. Here the authors show that mice with constitutive CD11c-specific CD40-signalling have altered CD103^+^ dendritic cell migration, reduced iTreg cell induction, and fatal colitis.

The immune system of the gut discriminates between invading pathogens and colonizing commensal bacteria. Specialized populations of intestinal cells integrate local signals to regulate and maintain a mutualistic relationship with the microbiota^1^. Failure to integrate this information into proper regulatory processes can lead to pathologies such as inflammatory bowel diseases, allergy or metabolic dysregulation.

Foxp3^+^ regulatory T (Treg) cells are important for such homeostatic balance by controlling immune responses^2^. Treg cells can be generated in the thymus from developing CD4^+^ thymocytes (nTregs), as well as by differentiation from mature peripheral CD4^+^ T cells to induced Tregs (iTregs), a process requiring transforming growth factor β (TGF-β)^3^. Germ-free mice have reduced Treg cell numbers^4^, a deficit that can be rescued by colonization with commensal bacteria^5^, suggesting that microbes cause colonic iTreg cell differentiation or expansion. iTreg and nTreg cells occupy distinct cellular niches, indicating a non-redundant role for iTreg cells to control mucosal homeostasis^6^. A large fraction of colonic Foxp3^+^ Treg cells is induced by the microbiota to express retinoic acid receptor-related orphan γt (RORγt)^7^,^8^, and the deletion of RORγt^+^ iTreg cells caused increased production of intestinal IL-17A and interferon-γ (IFN-γ) in one study^8^ or elevated type 2 helper T (Th2)-responses in another study^7^. Although both studies demonstrated the importance of RORγt^+^Foxp3^+^ iTregs to suppress T effector cells in the gut, the precise anti-inflammatory role of RORγt^+^Foxp3^+^ iTreg cells is unclear^9^.

Dendritic cells (DC) present commensal and dietary antigens to T cells. CD103^+^ DCs in the lamina propria (LP) of the intestine take up bacterial antigen efficiently from the gut lumen^10^ or from CX3CR1^+^ macrophages^11^ to induce the development of peripheral iTreg cells^12^,^13^. CD103^+^CD11b^+^ DCs are a major subpopulation of tolerogenic DCs, which can also induce Th17 cells^14^,^15^ or Th17 and Th1 cells upon activation with Toll-like receptor (TLR)-ligands^16^,^17^. CD103^+^CD11b^−^ DCs express high levels of aldehyde dehydrogenase (ALDH), TGFβ, integrin β_8_ and several other proteins necessary for induction of iTreg cells and gut homing^17^. By contrast, most CD103^−^ DCs in the LP express CD11b, have a phenotype similar to macrophages, and can prime IL-17-producing and IFN-γ-producing T cells in steady state without further stimulation^17^. Studies revealed precise roles of the distinct DC subsets showing that CD103^+^CD11b^−^ DCs migrating from LP to draining LN, but not sessile CD64^+^ monocyte-derived cells are essential for the induction of iTreg cells^18^.

The exact mechanisms controlling the functional switch between tolerogenic iTreg-inducing versus immunogenic CD103^+^ DCs is elusive. Pattern recognition receptors and inflammatory signals certainly have a function in functional DC-modulation; however, many microbial products are shared between commensal and pathogenic microorganisms, making them ambivalent signals for DC to induce tolerance or immunity. On the other hand, signals delivered by immune cells could also suppress iTreg-generation when immune responses are needed. CD40-signals can stop Treg-suppression of DCs^19^ and modulate CD103-expression by DCs^20^.

To further investigate the role of CD40-signalling, here we study external CD40-triggers and analyse transgenic mice expressing latent membrane protein 1 (LMP1)/CD40-molecules, inducing a constitutive active CD40-signalling in DCs. We show that CD40-signals cause few phenotypic changes in DCs. However, CD103^+^ DCs of the intestinal LP upregulate CCR7, migrate from the LP to mesenteric lymph nodes (mLNs) and rapidly die by apoptosis. Continuous CD40-signalling disables CD103^+^ DCs to induce RORγt^+^Foxp3^+^ iTreg cells and causes accumulation of IL-17A^+^IFN-γ^+^ Th17/Th1 T cells, breakdown of tolerance to gut microbiota, dysbiosis and fatal colitis. Our data describe CD40-triggering as a microbe-independent signal sufficient to modulate the tolerogenic properties of LP CD103^+^ DCs.

## Results

### CD40-induced migration of intestinal DCs to mLNs

**Figure 1:**
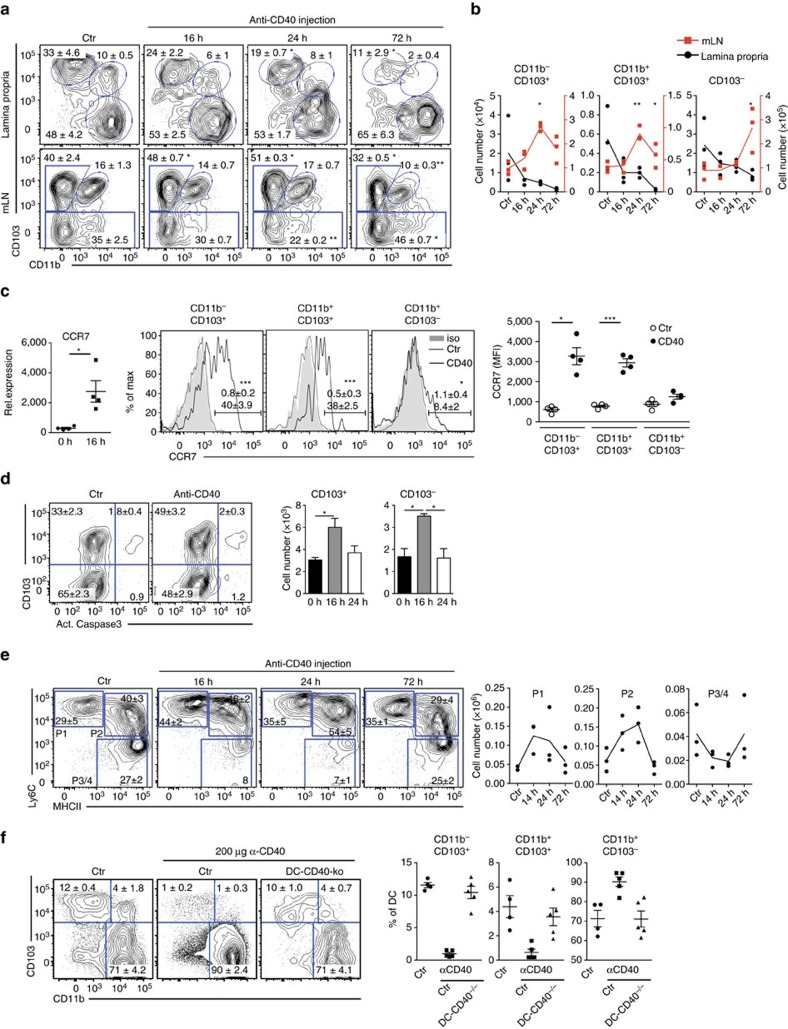
Injection of anti-CD40 antibody induces migration of DCs from LP to mLNs. (**a**) Representative FACS plots of DC subsets as differentiated by CD103 and CD11b in LP (gated on live CD45^+^MHCII^+^CD11c^+^CD64^−^ cells, Supplementary Fig. 1a) and mLNs (gated on live MHCII^+^CD11c^+^ cells, Supplementary Fig. 1b) at different time points after CD40 injection are shown. Numbers in the FACS plot indicate the percentages of cells in the respective subset. (**b**) Numbers of cells per DC subset from (**a**) LP (black) and mLNs (red) are shown. (**c**, left) CCR7 mRNA expression was analysed in CD11c^+^MHCII^+^ cells purified from the LP of control mice and 16 h after anti-CD40 injection. (central) CCR7 surface expression of distinct LP DC subsets was analysed in untreated control mice (Ctr) and mice treated with anti-CD40 mAb (iso, isotype control) 24 h after Ab-injection by flow cytometry. Numbers in histograms indicate per cent CCR7^+^ DCs in the respective gate (mean±s.e.m; upper number, Ctr; lower number, anti-CD40 injected). (right) Shown is the MFI of DCs. Distinct DC-subsets were gated as shown in **a**. (**d**) Representative FACS plots of active caspase3 in CD103^+^ and CD103^−^ DCs from mLNs (gated on live MHCII^+^CD11c^+^ cells, Supplementary Fig. 1b) 16 h after anti-CD40 mAb injection and untreated controls. Bar graphs show the number of cells positive for active caspase3 at different time points after anti-CD40 injection (*n*=3). (**e**) Distribution of macrophages within the `waterfall' staining (gated as shown in Supplementary Fig. 3a) at different time points after anti-CD40 injection shown as representative FACS plots and cell numbers. Shown is one representative of two experiments (*n*=3). (**f**) Control and DC-CD40^−/−^ animals were injected with an antibody (FGK45) and DC subsets analysed 3 days post injection. Dot plots and graphs show representatives of two (**a**–**d**,**f**) or three (**e**) independent experiments. Depicted is the mean±s.e.m. of (*n*=3; **a**,**b**,**d**–**f**) or *(n*=4; **c**) 8–10 wk old female mice of each strain per group. *P<0.05; **P<0.01; ***P<0.001; two-tailed unpaired *t*-test. MFI, mean fluorescence intensity.

Various signals have been identified that enable DCs to develop tolerogenic iTreg-inducing functions. Besides GM-CSF, RA and TLR2 signalling, also β-catenin-dependent signals, uptake of apoptotic DCs and PD-1 ligation may imprint Foxp3^+^ Treg induction (reviewed in ref. 21). In contrast, it is much less clear which signals abrogate Treg induction by DCs, for example in situations where induction of immunity is warranted. Besides microbial stimuli also CD40-signals can modulate the function of CD103^+^ DCs. For example, injection of anti-CD40 monoclonal antibodies (mAbs) can reduce the numbers of splenic CD103^+^ DC^20^. Yet, triggering of CD40 is known to induce incomplete maturation and increased survival of DCs^22^, which only become fully matured, when CD40-signalling is combined with a microbial trigger^23^,^24^. To investigate the influence of CD40-signalling on DCs *in vivo*, we injected anti-CD40 mAbs into C57BL/6 mice and analysed DC subsets in the LP and mLNs. All DC subsets in the LP are strongly reduced at 72 h post treatment, with a more prominent effect on both CD103^+^CD11b^−^ and CD103^+^CD11b^+^ DC subsets (Fig. 1a,b). To find out if the decreased DC numbers in LP would be due to apoptosis, we analysed DCs for presence of activated caspase 3, a marker for cell death. Both, CD103^+^CD11b^−^ and CD103^+^CD11b^+^ DCs in LP show no increased levels of activated caspase 3 (Supplementary Fig. 2). Therefore, most likely cell death is not the reason for disappearance of CD103^+^ DCs from LP upon anti-CD40 stimulation (Supplementary Fig. 2). In parallel, the frequencies and numbers of CD103^+^ DC subpopulations transiently increase in the mLNs with a peak accumulation at 24 h post injection (Fig. 1a,b), suggesting that CD40-ligation induces migration of DCs from the LP to mLNs. This interpretation is further strengthened by the finding that CD11c^+^MHCII^+^ DCs sorted from the LP after anti-CD40 treatment upregulate expression of mRNA for CCR7 (Fig. 1c), which is a requirement for migration of CD103^+^DCs from LP to mLNs^25^. Also, CCR7-surface levels are upregulated strongly by CD103^+^CD11b^−^ and CD103^+^CD11b^+^, but to a much lesser extent by CD103^−^ DCs (Fig. 1c, centre and right hand panel). Since the increase of CD103^+^DCs in mLNs is only transient, we investigated the fate of these cells and analysed DCs in mLNs for the presence of activated caspase 3 as a marker for apoptosis (Fig. 1d). Numbers of activated caspase 3^+^ CD103^+^ and CD103^−^ DCs briefly increase at 16 h post treatment, but are then comparable to controls, suggesting a transiently increased number of apoptotic cells (Fig. 1d). Also monocyte-derived macrophages are involved in immune homeostasis of the intestine^26^. We therefore analysed different macrophage subpopulations P1 – P4 as described previously^27^ (Supplementary Fig. 3a). This analysis showed that anti-CD40 injection also modifies the composition of macrophages in the intestine. The proinflammatory Ly6C^+^ subpopulations P1 and P2 transiently increase in population size, while the frequencies of Ly6C^−^MHCII^+^ subpopulation P3/4 decrease relatively to the other subpopulations, but remain relatively unaltered in total cell numbers (Fig. 1e). However, in contrast to DCs, macrophages of the LP do not migrate to the mLNs, as previously published^28^.

Injection of anti-CD40 mAb leads to severe liver inflammation and rapid increase in serum levels of alanine-aminotransferase (Supplementary Fig. 3b)^29^. Moreover, strong increase of inflammatory cytokines such as MCP1, TNF, IFN-γ and IL-6 can be detected in the serum as early as 16 h post injection (Supplementary Fig. 3c). Therefore, injection of anti-CD40 mAbs causes secondary effects, which could influence or even be responsible for triggering CD103^+^ DCs to migrate to mLNs. To investigate if the direct signal of CD40-crosslinking on DCs rather than the concomitant inflammatory signals would induce CD103^+^ DCs to migrate to the mLNs, we tested mice that lacked CD40 specifically on CD11c^+^ DCs (CD11c-Cre x CD40^fl/fl^). Here, CD40mAb-treatment does not induce migration of CD103^+^ DCs from the LP, arguing for the need of CD40-crosslinking directly on DCs rather than secondary effects for induction of DC-migration to mLN (Fig. 1f).

These findings suggest that CD40-triggered DCs leave the LP and migrate to mLNs, where they eventually die by apoptosis.

### DCs in DC-LMP1/CD40 mice have continuous CD40-signalling

Although the above experiments suggest that CD40-signalling causes DCs to leave the LP, we could not exclude an influence of inflammatory stimuli, which have been previously reported to augment effects of CD40-ligation on DCs^23^,^24^,^30^. To analyse the functional role of CD40-signalling on iTreg-inducing DCs in absence of other inflammatory signals *in vivo*, we generated mice where CD11c^+^ DCs receive a constitutive, ligand-free CD40 signal. To this end, we generated DC-LMP1/CD40-mice by breeding CD11c-Cre mice^31^ to the previously published LMP1/CD40^flStop^ mice^32^, which express a loxP-flanked stop-codon-protected LMP1/CD40 chimeric protein from the Rosa26 locus. The LMP1/CD40-fusion protein consists of the signalling domain of CD40 and the transmembrane domain of LMP1. It has been previously shown that expression of this chimeric LMP1/CD40 fusion protein in B cells leads to constitutive signalling via noncanonical NF-kappaB signalling pathway and the MAPK Erk and Jnk resulting in lymphomagenesis^32^. The CD11c-Cre strain has been used to direct Cre-expression to intestinal DCs in previous studies^33^. As expression of the LMP1/CD40 fusion protein in DCs was too low to be detected by western blot analysis or flow cytometry, we analysed the expression of Cre in intestinal DCs and macrophages indirectly with the help of tdRFP reporter mice, which express loxP-flanked stop codon-protected RFP from the Rosa26 locus^34^. This analysis showed that Cre was expressed in CD64^−^CD11c^+^MHCII^+^ DCs, but not in the majority of macrophages (Supplementary Fig. 3d). Here only macrophages of the MHCII^+^Ly6C^low^ P3/P4 subtype were partially RFP^+^. Therefore, LMP1/CD40-fusion protein expression was largely restricted to DCs and to MHCII^+^Ly6C^low^ CD11c^+^ macrophages in the intestine.

### Expression of LMP1/CD40 in DCs causes fatal colitis

**Figure 2:**
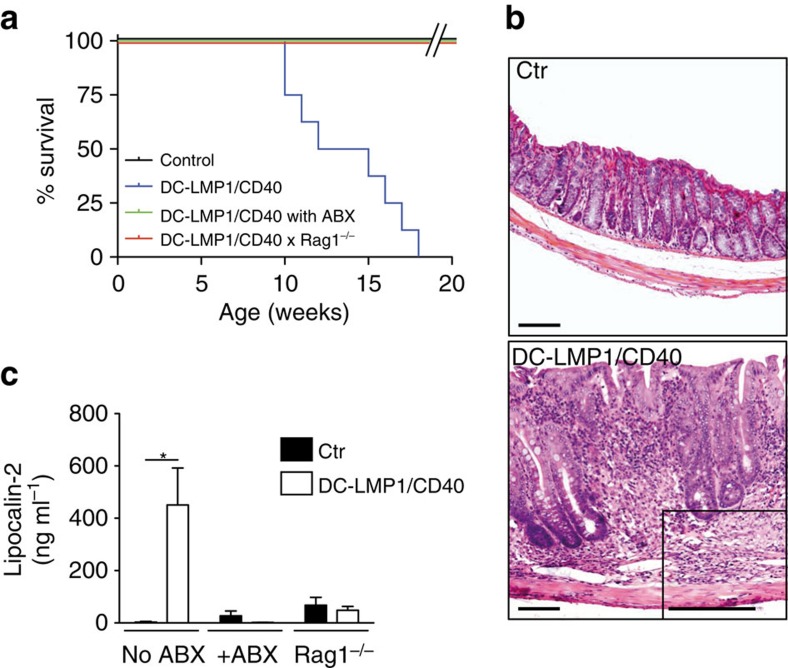
DC-LMP1/CD40 animals develop fatal spontaneous colitis. (**a**) Kaplan–Meier plot showing the survival of control and untreated or ABX-treated DC-LMP1/CD40 animals on C57BL/6 and Rag1^−/−^ background (*n*≥6). (**b**) DC-LMP1/CD40 mice display severe colitis with thickening of the colon mucosa, extensive proprial infiltration of mixed inflammatory mononuclear cells, loss of crypts and reduction of goblet cells. Paraffin sections, inset to DC-LMP1/CD40: GMA/MMA section, HE-staining. Scale bars, 100 μm. (**c**) Levels of faecal lipocalin-2 as measured by ELISA in 8–10-week-old mice (*n*≥3 per group). Graphs show representatives of two (**a**) or three (**c**) independent experiments. Depicted is the mean±s.e.m. of (*n*=8; **a**) or *(n*=6; **c**) individual 8–10–week-old female animals per group. *P<0.05; two-tailed unpaired *t*-test.

The lifespan of DC-LMP1/CD40 mice is drastically shortened to 10–20 weeks (Fig. 2a). Mouse pathology shows marked thickening of the colon mucosa, extensive LP infiltrates of mixed inflammatory mononuclear cells (including lymphocytes, plasma cells, macrophages and neutrophils), loss of crypts, reduction of goblet cells, as well as focal cryptitis and ulceration (Fig. 2b). In addition, levels of faecal lipocalin-2, a biomarker for intestinal inflammation^35^, increase significantly in DC-LMP1/CD40-mice (Fig. 2c). In contrast, histopathological examination of spleen (Supplementary Fig. 4a) and other organs of DC-LMP1/CD40 transgenic mice did not reveal noticeable pathological alterations.

To test if the development of colitis was dependent on T and B cells or commensal bacteria, we bred DC-LMP1/CD40-mice to T and B cell deficient Rag1^−/−^ mice or treated DC-LMP1/CD40-mice with a mixture of antibiotics (ABX), respectively. DC-LMP1/CD40 x Rag1^−/−^ show long-term survival like non-transgenic control littermates (Fig. 2a) and no thickening of the mucosa (Supplementary Fig. 4b). The levels of lipocalin-2 as a marker for gut inflammation are similar to those of healthy Rag1^−/−^ mice (Fig. 2c). Similarly, the reduction of commensal bacteria by ABX-treatment prevents onset of colitis (Fig. 2a) as well as lesions of the colon mucosa (Supplementary Fig. 4b). Overall, this data indicates that CD40-signalling in DCs was sufficient to induce fatal colitis, which depends on both, lymphocytes and the presence of high luminal loads of commensal bacteria.

### DC-LMP1/CD40-mice have reduced frequencies of CD103^+^ DCs

**Figure 3:**
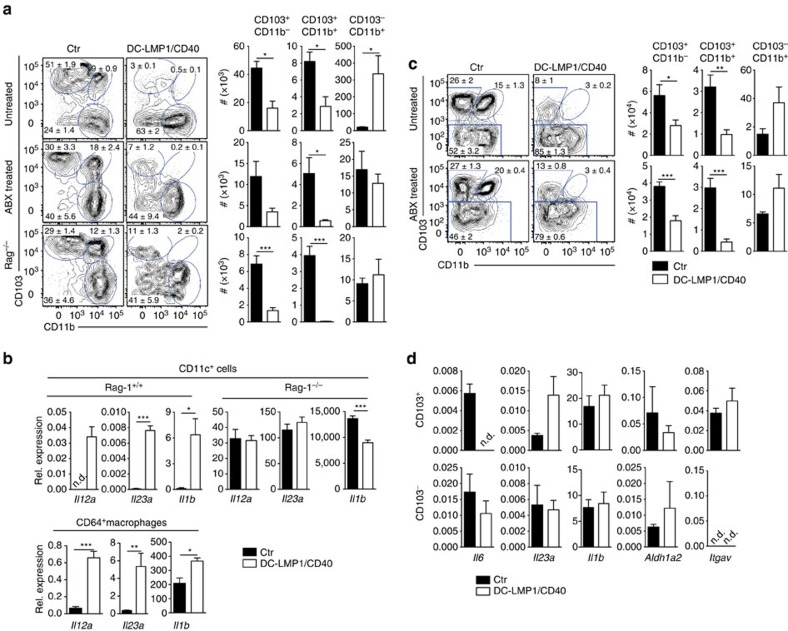
CD103^+^ DCs are strongly reduced in the LP and mLNs of DC-LMP1/CD40 animals. DC subsets in the LP (**a**,**b**) or mLNs (**c**,**d**) were analysed as shown in Supplementary Fig. 1a,b with gates on live, CD45^+^CD11c^+^MHCII^+^(CD64^−^) cells from control, ABX-treated or Rag1^−/−^ mice. Representative FACS-plots are shown, numbers indicate frequency of DC subsets and bar graphs show absolute numbers per colon. (**b**) RNA from MACS- or FACS-purified (upper and lower panel, respectively) CD11c^+^ cells was analysed by qPCR (DC-LMP1/CD40 mice, controls) or the nanostring platform (DC-LMP1/CD40xRag1^−/−^ mice, Rag1^−/−^ controls). (**c**) Representative FACS plots (left) and statistics (right) of DC subsets in the mesenteric lymph node of untreated DC-LMP1/CD40 (top) and ABX-treated DC-LMP1/CD40 animals in comparison with controls. Cells are gated on live CD11c^+^MHCII^+^. (**d**) CD103^+^ and CD103^−^ DCs were sorted from mLNs (live CD11c^+^MHCII^+^CD64^−^) of control and DC-LMP1/CD40 animals and qPCRs for indicated genes were performed using mRNA isolated from these cells. Data are normalized to Ubiquitin expression (*n*=3–5). Dot plots and graphs show representatives of two (**b**,**d**) or three (**a**,**c**) independent experiments. Depicted is the mean±s.e.m. of (**a**, *n*=6; **b**, *n*=6; **c** ‘untreated' *n*=10; **c** ‘ABX treated' *n*=5; and **d**, *n*=5) individual 8–10-week-old female animals per group. *P<0.05; **P<0.01; ***P<0.001; n.d., not detectable; two-tailed unpaired *t*-test.

To compare the effects mediated by the LMP1/CD40-fusion protein to anti-CD40-injection (Fig. 1), we next analysed the DC-subsets of the colon (Fig. 3a). In DC-LMP1/CD40 animals the frequencies and cell numbers of both CD103^+^CD11b^−^ and CD103^+^CD11b^+^ DC subsets in the LP are strongly reduced (Fig. 3a). In contrast, CD103^−^CD11b^+^ DCs are present in higher amounts (Fig. 3a). To test if these changes are caused by the CD40-fusion protein or by secondary inflammatory effects due to colitis (Fig. 2a,c), we also analysed mice treated with ABX or on Rag1^−/−^ background (Fig. 3a), which are free of colitis (Fig. 2 and Supplementary Fig. 4b). Both groups show a similarly significant reduction of CD103^+^ DC subsets, while CD103^−^CD11b^+^ DCs are unchanged in numbers (Fig. 3a). This finding indicates that CD103^−^CD11b^+^ DCs seem to be less susceptible to CD40-signal-induced migration as compared with CD103+ DCs, either induced by mAb (Fig. 1a,b) or by the LMP/CD40 transgene (Fig. 3a). Therefore, in contrast to the increase in CD103^−^ DCs, which is most likely caused by secondary inflammatory effects, the loss of CD103^+^ DCs from LP is intrinsically caused by the LMP1/CD40-transgene.

To find out if the expression of LMP/CD40 transgene would induce DC-maturation, we analysed surface markers and cytokine expression. However, we could not detect substantial differential expression of surface markers such as CD86 (Supplementary Fig. 5) by DCs from DC-LMP1/CD40 mice as compared with control DCs. However, all DCs from DC-LMP/CD40 mice consistently show lower surface expression levels of MHC II and weak upregulation of CD80 in LP and mLNs (Supplementary Fig. 5). We next isolated cells to analyse cytokine gene expression. CD11c^+^ cells (DCs and macrophages) purified from inflamed colon of DC-LMP1/CD40-mice show strongly elevated expression of genes *Il23a* (encoding for IL-23p19), *Il12a* (encoding IL-12p35) and *Il1b* (encoding IL-1β) (Fig. 3b, upper panel). In contrast, CD11c^+^ cells from DC-LMP1/CD40xRag1^−/−^-mice that do not develop colitis (Fig. 2a) do not express elevated levels of *Il23a*, *Il12a*, *il1b* (Fig. 3b). This suggests that cytokine induction in CD11c^+^ cells from DC-LMP1/CD40-mice is a secondary effect of inflammation, rather than being induced by transgene expression alone.

We next analysed effects of spontaneous colitis in the DC-LMP1/CD40-model on monocyte-derived macrophages of the colon and characterized CD64^+^ cells of the LP (Supplementary Fig. 6) as described previously^27^(Supplementary Fig. 3a). In inflamed colon of DC-LMP1/CD40-mice the total cell numbers of all CD64^+^ macrophage subpopulations as characterized by differential Ly6C and MHCII expression are increased (Supplementary Fig. 6), similar to macrophages in the colon of anti-CD40 mAb-treated animals (Fig. 1). However, in non-inflamed colon of ABX-treated DC-LMP1/CD40-mice, numbers and frequencies of macrophages are normal (Supplementary Fig. 6). This data suggests that changes in macrophage numbers in the colon of DC-LMP1/CD40-mice are rather caused by secondary effects of colitis, but are not intrinsically due to LMP1/CD40-expression in some CD11c^+^ macrophages. While LP-derived DCs (CD11c^+^MHCII^+^CD64^−^) in DC-LMP/CD40-mice could not be analysed due to low numbers, we sorted CD11c^+^MHCII^+^CD64^+^ macrophages and tested for cytokine gene expression (Fig. 3b). CD11c^+^MHCII^+^CD64^+^ macrophages from LP of DC-LMP/CD40-mice show significantly elevated levels of inflammatory *Il23a*, *Il12a* and *Il1b* (Fig. 3b). This data suggests that macrophages, which increase massively in T cell dependent colitis models^27^, contribute to colitis also in DC-LMP1/CD40-mice. However, this effect is not transgene intrinsic, but secondary, as ABX-treatment inhibits MP-accumulation (Supplementary Fig. 6) and lack of T- and B cells in Rag1^−/−^-mice abrogates generation of inflammatory cytokines (Fig. 3b).

In mLNs the frequencies and absolute numbers of CD103^+^ DCs are similarly reduced, both, under inflammatory conditions (Fig. 3c, upper panel) as well as in ABX-treated mice, which do not develop colitis (Fig. 3c, lower panel), suggesting that CD40-signalling induces robust DC-migration from LP to mLNs, but constitutive signalling in DC-LMP/CD40-mice would not lead to transient accumulation of DCs in mLN, as observed in acute treatment with anti-CD40 mAbs (Fig. 1b).

We next sorted CD103^+^ and CD103^−^ DCs from the mLNs of DC-LMP1/CD40-mice with colitis for expression analysis. Cytokine genes *Il6*, *Il23a* and *Il1b* are expressed similarly in DCs from transgenic and control mice (Fig. 3d). Although there is a tendency for *Il23a* to be upregulated in CD103^+^ DCs, this does not reach significance. Also the expression of ALDH (*Aldh1a2*) that is important for generation of Treg-inducing retinoic acid^12^ is not altered (Fig. 3d). Similarly, integrin αv (*Itgav*) that forms integrin αvβ8 together with β8 and specifically equips CD103^+^ DCs with TGF-β-activating capacities for Treg induction^36^ are not differentially expressed (Fig. 3d). This data indicates that expression of genes important for Treg induction are not significantly altered in DCs from DC-LMP1/CD40-mice.

This data indicates that continuous CD40-signalling does not alter cytokine expression of CD103^+^ DCs, which are important for Treg induction. Taken together, our data from CD40-injection experiments and DC-LMP1/CD40 mice suggest that CD40-signals induce CD103^+^ DCs to migrate to draining mLNs, where they die by apoptosis (Fig. 1d). As the signal in LMP1/CD40-transgenic mice is continuous, but not inducible like acute antibody-injection, we can neither observe transient accumulation nor increased apoptosis of CD103^+^ DCs in mLNs. As a net result, constitutive CD40 stimulation leads to sustained shifts in DC subset composition with strong reduction of CD103^+^ DCs and increased numbers of CD103^−^ DCs.

### DC-LMP1/CD40-mice lack RORγT^+^ iTreg cells

**Figure 4:**
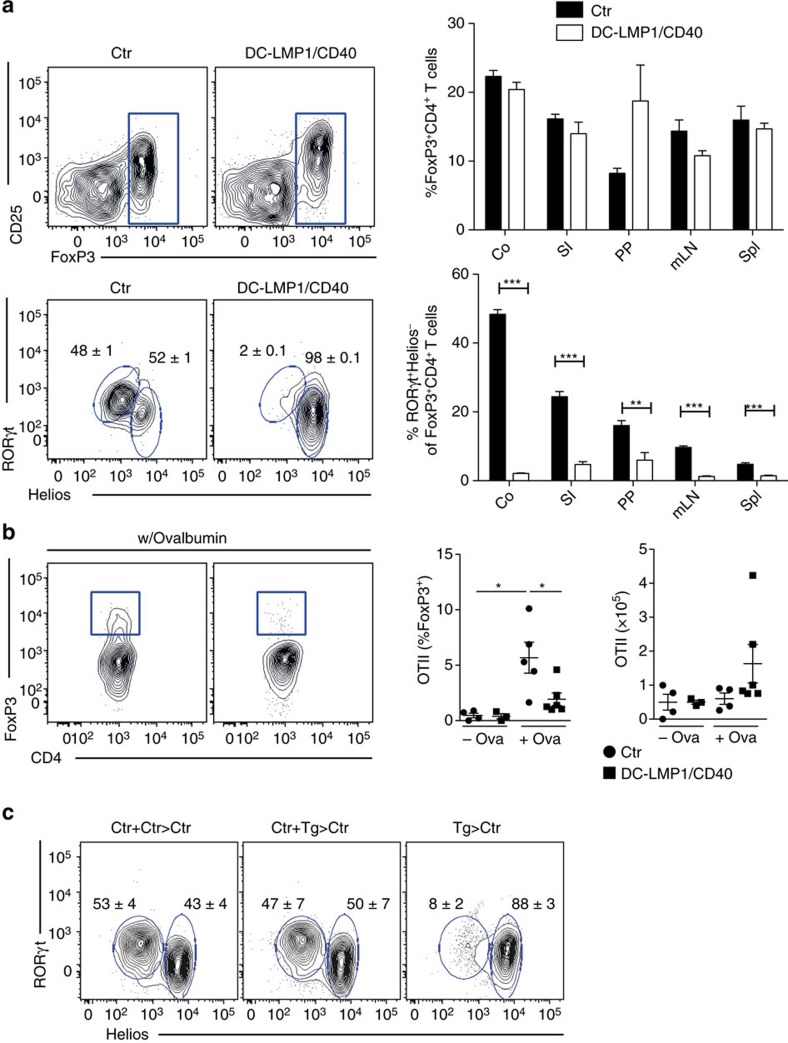
iTreg induction is severely impaired in DC-LMP1/CD40 animals. (**a**) Single-cell suspensions of different organs were analysed for Tregs as shown in Supplementary Fig. 1e). The upper panel shows representative FACS plots of Foxp3 expression in CD3^+^CD4^+^ T cells from colon LP. The percentage of Tregs in the CD4^+^ T-cell compartment of the indicated organs is shown in the right panel. The lower panel shows a representative FACS plot and statistics of nTreg (RORγt^−^Helios^+^) and iTreg (RORγt^+^Helios^−^) distribution within Foxp3^+^ T cells. (**b**) OTII cells were transferred into Ctr and DC-LMP1/CD40 animals, which were treated with ABX for 3 weeks, in order to eliminate inflammation and were then fed with OVA ad libitum. Expression of Foxp3 was analysed in OTII cells (live CD4+CD90.1+ cells) in mLNs after 5 days. Percentages and total cell numbers are shown as mean±s.e.m. of one representative of two experiments (*n*=3) in the graphs. (**c**) iTreg induction was analysed in single-cell suspension of colonic LP of BM chimeras reconstituted with the indicated BM (Ctr+Ctr>Ctr: 50%Ctr-BM(CD45.1)+50%Ctr-BM(CD45.2)>Ctr(CD45.1); Ctr+Tg>Ctr: 50%Ctr-BM(CD45.1)+50%DC-LMP/CD40-BM(CD45.2)>Ctr(CD45.1). Tg>Ctr: 100%DC-LMP1/CD40-BM(CD45.2)>Ctr (CD45.1)). Percentage of nTregs and iTregs was determined as in **a**. Shown is one representative result of two experiments with similar outcome (*n*=3). Dot plots and graphs show representatives of two independent experiments (**b**,**c**) or pooled results of two experiments (**a**). Depicted is the mean±s.e.m. of (a, *n*=6; b, *n=3–6;* c, *n*=3) individual 8–10-week-old female animals per group. *P<0.05, ***P<0.001; two-tailed unpaired *t*-test.

In contrast to the short-term application of a CD40-specific mAb, the DC-LMP1/CD40 model allowed us to investigate the effect of long-term reduction of CD103^+^ DCs. As CD103^+^ DCs have been postulated to induce Tregs^12^,^13^, we next investigated Foxp3^+^ cells in different organs of DC-LMP1/CD40 mice (Fig. 4). However, we did not find any differences in the frequency of Foxp3^+^CD4^+^ Tregs in tissues and organs of DC-LMP1/CD40 in comparison with control mice (Fig. 4a, upper panel). When we further differentiated between nTreg and iTreg by using the markers Helios^+^RORγt^−^Foxp3^+^ for thymus-derived nTregs and Helios^−^RORγt^+^Foxp3^+^ for peripherally induced iTregs, we found between 50 and 60% of the Tregs in colonic LP of control animals to be RORγt^+^Foxp3^+^ iTregs (Fig. 4a, lower panel). However, this population is virtually absent in DC-LMP1/CD40 animals (Fig. 4a, lower panel). Also, iTregs in other tissues of DC-LMP1/CD40-mice are strongly reduced (Fig. 4a, lower panel). To test if iTreg-induction is abolished in DC-LMP1/CD40-mice, we administered chicken ovalbumin (OVA) in the drinking water and monitored adoptively transferred naive OVA-specific TCR-transgenic CD4^+^ OTII T cells for induction of Foxp3 (Fig. 4b). To avoid secondary effects due to the inflammatory environment between controls and DC-LMP1/CD40-mice, these experiments were performed in colitis-free ABX-treated mice. Oral administration of antigen very strongly induces tolerance^37^ and mediates conversion of naive CD4^+^ T cells into iTregs^7^. In contrast to control animals, where Treg induction can be readily observed, DC-LMP1/CD40 animals fail to induce peripheral iTregs (Fig. 4b). However, OTII cells also expanded in DC-LMP1/CD40-mice, as tested by determination of total numbers of expanded CD4^+^ OTII cells (Fig. 4b). Taken together, this data suggests that in DC-LMP1/CD40 mice antigen-specific CD4 T cell priming does occur in mLNs, but induction of iTregs is defective.

Next, we wanted to determine if lack of iTreg was dominant or if it could be rescued by the presence of normal numbers of CD103^+^ DCs. To this end we generated bone marrow (BM) chimeras. When irradiated wt mice received BM from DC-LMP1/CD40-mice they behaved similar to non-irradiated DC-LMP1/CD40-mice, since they cannot support development of iTregs (Fig. 4c). In contrast, mixed chimeras, which were reconstituted with a 1:1 mixture of BM from wt- and DC-LMP1/CD40-mice, show normal frequencies of intestinal iTregs similar to chimeras reconstituted with BM from wt-mice only (Fig. 4c). Analysis of the DC-subpopulations in the chimeras confirmed the relatively lower CD103^+^ DC numbers as compared with CD103^−^ DCs in BM of DC-LMP1/CD40-origin in comparison with controls (Supplementary Fig. 7). This data suggests that replenishment of the CD103^+^ DC-compartment in LP and mLNs is sufficient to induce Helios^−^RORγt^+^Foxp3^+^ iTreg and protect from colitis.

### LMP1/CD40-mice have dysregulated intestinal tolerance

**Figure 5:**
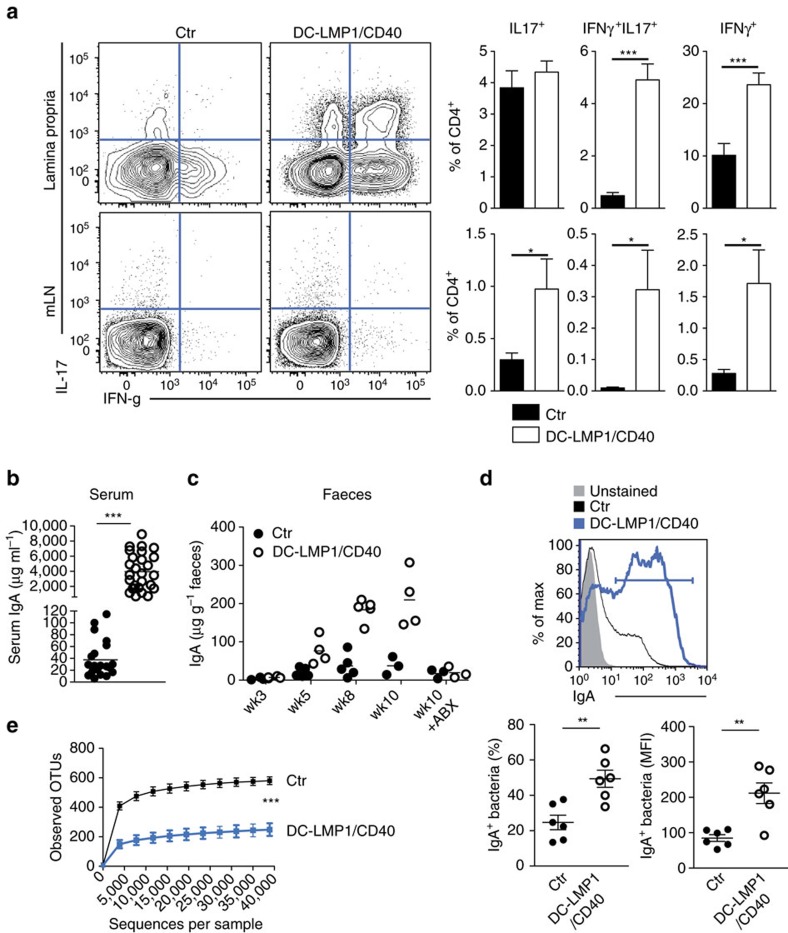
Breakdown of B- and T-cell tolerance. (**a**) T-cell functionality was analysed by stimulating single-cell suspensions with PMA/Ionomycin and subsequently staining cells intracellular for the production of IL-17 and IFNγ as shown in Supplementary Fig. 1c, d. Shown are representative FACS plots for the respective organs as well as pooled statistics from more than five experiments (*n*=14–18). (**b**) Binding of serum antibodies from 8-week-old mice to cecal content was analysed in an ELISA experiment using anti-mouse IgA-specific antibodies as detection antibodies. (**c**) Overall antibody amount specific for cecal content was measured as in **b** (*n*=4, one out of three experiments with similar results is shown). (**d**) IgA-coated fecal bacteria from control and DC-LMP1/CD40 animals. Faeces homogenates were stained with anti-mouse IgA and analysed by flow cytometry as shown in Supplementary Fig. 1f. Data shows one representative histogram (upper panel) with percentage (lower left panel) or MFI (lower right panel) of IgA^+^ bacteria (two pooled experiments, *n*=6). (**e**) Differences in alpha diversity of co-housed 8-week-old control and DC-LMP1/CD40 animals. On the basis of 16S rRNA gene sequencing of the V3-V4 regions the number of observed OTUs on the *y* axis versus the number of sequences per sample on the *x* axis are shown as rarefaction curve. Data indicates mean±s.d. (*n*=5). Statistical analysis was done with a two-tailed unpaired Student's *t*-test. Dot blots and graphs show representatives of three (**b**,**c**) or five (**a**) independent experiments. Depicted is the mean±s.e.m. of *(n*=4; **b**,**c**), (*n*=5; **e**), (*n*=18, **a**) individual 8–10-week-old female animals per group. (**d**) shows pooled data from two individual experiments with *n*=6 animals. *P<0.05; **P<0.01; ***P<0.001; two-tailed unpaired *t*-test. MFI, mean fluorescence intensity.

Since absence of colitis in DC-LMP1/CD40xRag^−/−^ mice (Fig. 2 and Supplementary Fig. 4b) suggested an involvement of B and/or T cells in colitis development in DC-LMP1/CD40-mice, we next characterized T and B cells further. DC-LMP1/CD40-mice show significantly increased frequencies of IL-17A^+^ Th17 cells, IL-17A^+^IFN-γ^+^ Th17/Th1 cells and IFN-γ^+^ Th1 in mLNs and higher levels of Th17/Th1 and Th1 cells in LP as compared with control mice (Fig. 5a). To test B cell responses, we analysed titres of serum antibodies specific for commensal antigens and found increased levels of commensal-specific IgA in DC-LMP1/CD40 animals (Fig. 5b). The specific IgA-levels in serum increase with the age of the mice (Fig. 5c), but are absent in ABX-treated mice (Fig. 5c). In contrast to co-housed control mice, a higher percentage of intestinal microbiota of DC-LMP1/CD40 mice is IgA-coated (Fig. 5d upper and lower left panel) and bound more IgA per microbe (Fig. 5d, lower right panel). Amplicon sequencing of the 16S rRNA gene regions V3-V4 revealed a strong and statistically significant decrease in the number of observed operational taxonomic units (OTUs) in DC-LMP1/CD40-mice as compared with co-housed control littermates (Fig. 5e), indicating a severe decrease in microbiota α-diversity, a general sign of dysbiosis. Therefore, LMP1/CD40-induced migration and reduction of CD103^+^ DCs led to loss of Helios^−^RORγt^+^Foxp3^+^ iTreg, breakdown of intestinal tolerance with higher numbers of pathogenic IL-17A^+^IFN-γ^+^ Th17/Th1 and IFN-γ^+^ Th1 CD4 T cells and commensal-specific antibodies.

## Discussion

Here, we report that acute and constitutive CD40-triggering of intestinal DCs induces their robust migration from LP to the mLNs leading to strong reduction of CD103^+^CD11b^−^ and CD103^+^CD11b^+^ DCs. Such changes in DC-homeostasis were accompanied by strongly reduced frequencies of Helios^−^RORγT^+^ iTreg and increased LP IFN-γ^+^ Th1 and IL-17A^+^IFN-γ^+^ Th17/Th1 cells. The latter can be found in the inflamed intestine of both humans and mice^38^,^39^ and are required for the pathogenesis of colitis^40^. However, ‘classical' Th17 cells, which have protective roles in tissues and contribute to barrier integrity^41^, were not altered in the LP.

A likely explanation for the increase in Th17/Th1- and Th1-cells and intestinal pathology is the very strong decrease of Helios^−^RORγT^+^ iTreg-frequencies, suggesting a defective ability of colonic Tregs to regulate inflammation. It has been previously reported that the gradual numerical decrease of Foxp3^+^ Tregs by injection of titrated amounts of diphteria toxin into Foxp3^DTR^-mice did augment Th1 and Th17 cells^42^ and that regulation of Treg numbers is MHCII^+^ DC-dependent^43^. As CD103^+^ DCs have the unique capacity to induce iTreg cells from naive CD4 T cells^12^,^13^,^18^, their strongly reduced frequencies in DC-LMP1/CD40-mice result in less efficient iTreg induction. This reduction is a recessive effect, as the presence of wt CD103^+^ DCs in mixed chimeras could reconstitute iTregs and protect from colitis. Similar to mice where Helios^−^RORγT^+^ iTreg were depleted genetically^7^,^8^, inflammatory T-cell responses were increased in the intestine, although overall frequencies of Foxp3^+^ Treg cells were not altered, suggesting a compensation by increased frequencies of Helios^+^RORγT^−^ nTreg cells. In line with our results, RORγT^+^ iTreg cells have been recently shown to more efficiently suppress colitis than their RORγT^−^ nTreg counterparts in a transfer colitis model^44^. It has been shown that nTreg would fail to fill the niche of iTreg^6^ because of their largely non-overlapping TCR repertoires^45^. In fact, despite the relative increase of nTreg, they could not functionally ‘replace' iTregs to regulate T-cell-mediated inflammation and colitis in DC-LMP1/CD40-mice.

DC-LMP1/CD40-mice develop colitis without further experimentally induced challenges or acute infections. In several other homeostatic models, where CD103^+^CD11b^+^ DCs were either genetically removed either alone or were depleted together with CD103^+^CD11b^−^ DCs^14^,^15^,^46^,^47^ homeostatic generation of IL-17A^+^ Th17 cells was reduced and mice did not develop colitis spontaneously. In contrast Th17 cells were not altered in LP of DC-LMP/CD40 mice, while Th17/Th1 and Th1 cells were strongly increased in the model of the present study. A major difference between DC-LMP/CD40 mice and the DC-ablation models published previously, is the deletion of CD103^+^ DCs, which are especially equipped to gather microbial and non-microbial products in the LP from the intestinal lumen^10^,^48^ or from CX3CR1^+^ macrophages^11^. Lack of CD103^+^ DCs might abolish antigen-acquisition and -transport as well as priming of antigen-specific CD4 T cells in the draining mLNs. In contrast to Th17 cells, the generation of intestinal iTregs from naive T cells depends on TCR-recognition of antigen/MHCII-complexes, as mice expressing a single TCR, but no cognate antigen^49^, or mice with MHCII-deficient APC^50^,^51^ cannot induce intestinal iTregs, but accumulate nTregs. Therefore, in contrast to other models, CD40-triggering does not deplete CD103^+^ DCs, but rather enforces their migration from the LP to the mLNs and antigen-transport is not abolished. This view is supported by the fact that OTII cells proliferated in OVA-fed DC-LMP1/CD40 mice in an antigen-specific fashion but failed to generate normal levels of iTregs. Other studies identified CD103^−^CD11b^+^ DCs as producers of IL-12p40 in the steady state^52^, enabling them to induce Th17 and Th1 cells even in absence of additional stimulation^17^. Therefore, migratory CD103^+^ DC might bring gut luminal antigens to the mLNs for presentation to CD4 T cells, either directly or upon transfer to other DCs. Such presentation might result in generation of Th17 cells, but only inefficient iTreg induction, a dysbalance that may lead to inflammatory colitis in DC-LMP1/CD40 mice. Alternatively, luminal antigens may reach the mLN by cell-independent pathways as described previously for OVA^53^ and then the composition of the DC-subsets able to differentially present antigen might determine the degree naive antigen-specific CD4 T cells differentiate into iTreg of CD4 effector cells.

IL-23 promotes inflammatory bowel disease^54^,^55^. However, neither CD103^+^ nor CD103^−^ DCs produced significantly elevated IL-23 levels in DC-LMP1/CD40-mice. The source of IL-23 apparently varies depending on the homeostatic or inflammatory models studied. For example, during acute intestinal inflammation CD103^+^CD11b^+^ DCs were the non-redundant source of IL-23 necessary for anti-*Citrobacter rodentium* responses^56^ and produced IL-23 upon TLR 5 stimulation with flagellin^52^. However, CD103^+^CD11b^+^ DCs have also been reported to be dispensable for *C. rodentium* protection^47^ and CX3CR1^+^ mononuclear phagocytes were the more critical IL-23-source^57^. Also during acute infection and IL10R blockade CD103^+^CD11b^−^ DCs were dispensable for IL-23 driven inflammatory pathology, while MHCII^+^ monocytes were the highest IL-23-producers^58^. As numbers of CD64^+^ monocytes increased during the onset of colitis in DC-LMP1/CD40-mice and CD64^+^CD11c^+^MHCII^+^ monocytes/macrophages, but not CD64^−^CD11c^+^MHCII^+^CD103^+^ DCs were strong producers of IL-12 and IL-23, it is likely that macrophages or blood-recruited monocytes sustain the inflammatory response also in DC-LMP1/CD40-mice, similar to other models of colitis^58^.

Due to the fact that our model is constitutive, but not inducible, it is difficult to precisely determine what actually initiates the inflammatory response, and which factors and cell types do rather sustain it, once it is established. Treatment with ABX could completely neutralize intestinal pathology, with normal frequencies of IL-17A- and IFN-γ-producing T cells and normal levels of commensal-specific antibodies. The fact that CD103^+^ DCs were diminished also in ABX-treated mice argues for an intrinsic, microbiota- and inflammation-independent effect of CD40-signalling in this DC-subset, which is in marked contrast to CD64^+^ macrophages and CD103^−^ DCs, that were present at normal levels. The increase of these populations in untreated DC-LMP1/CD40-mice can therefore be considered as a secondary CD40-independent effect. However, the reasons for the relative inert behaviour of CD103^−^ DCs to anti-CD40 mAb treatment or LMP1/CD40-signalling are currently not clear. The fact CD103^−^ DCs do express and can upregulate endogenous CD40 and do express the LMP1/CD40-transgene suggest that CD40-signalling might be wired differently in CD103^−^ DCs as compared with CD103^+^ DCs.

Taken together, we present a novel intrinsic model of colitis, where the migration of CD103^+^ DCs is induced, causing their numerical reduction by apoptosis in the draining mLNs. This leads to abnormally low frequencies of iTregs, breakdown of intestinal tolerance manifested by high frequencies of inflammatory IL-17A- and IFN-γ-producing T cells and high titres of commensal-specific IgA accompanied by commensal dysbiosis as results of severe colitis. These results show the importance of homeostatic distribution of intestinal DC-subpopulations for the maintenance of intestinal tolerance.

Our data fit with an intriguing model where CD40L-CD40 interaction between activated CD4^+^ T cells and DCs is important to counteract the generation of iTreg or their suppressive activity in order to boost immune responses. This mechanism has previously been shown to inhibit Treg activity, which caused premature contraction of influenza-specific CD8^+^ T cells in the late phase of response to infection^59^. Accordingly, CD40L-expressing activated T cells, NK cells as well as several other cell types^60^ would be able to temporarily shut down the tolerogenic properties of intestinal steady-state homeostasis by manipulating/removing iTreg-inducing intestinal CD103^+^ DCs. On the other hand, this mechanism might also sustain chronic type autoimmune diseases by continuous obstruction of Treg induction. Further work will be necessary to unravel if such a mechanism can contribute to rapid amplification of intestinal immune responses.

## Methods

### Mouse strains

DC-LMP1/CD40 mice were generated by crossing CD11cCre mice^31^ with LMP1/CD40^flstop^ mice^32^. LMP1/CD40 mice have been backcrossed onto the C57BL/6 background for at least 10 generations. To analyse transgene expression/Cre activity we crossed Gt(ROSA)26Sor^tm1Hjf^ (ref. 34) to CD11cCre animals. The resulting strain is called CD11cCrexRFP^flStop^. DC-CD40-ko mice were generated by crossing CD11cCre mice^31^ with CD40^fl^ -mice, which carry loxP-sites before CD40 exon2 and after CD40 exon3. Cre-mediated recombination by CD11c-Cre mice removes the ‘loxed'exons 2 and 3 of CD40, rendering a non-functional CD40 peptide (Lutgens *et al*., personal communication). Mice were analysed in sex and age-matched groups of 8–10 weeks of age. Littermate animals were used as control in a non-randomized, non-blinded fashion. The SPF-status of the facility was tested according to the Federation for Laboratory Animal Science Associations (FELASA) recommendations. Animal experiment permissions were granted by animal ethics committees Regierung von Oberbayern, Munich, Germany and Organismo preposto al benessere animale di Universita di Ferrara, Italy. All mice were bred and maintained at the animal facility of the Institute for Immunology, Ludwig-Maximillians-Universität München and the Department of Life Sciences and Biotechnology, University of Ferrara.

### Single-cell preparations

Single-cell suspensions of splenocytes and lymph nodes were prepared by meshing organs through a 100 μM cell strainer. Where necessary, red blood cells were lysed using ACK buffer for 5 min at room temperature. Number of living cells was determined using CASY Counter (OMNI Life Science). To analyse cells from the LP, colon was taken from a mouse, faecal content removed, the colon opened longitudinally and cut into ca. 5 mm big pieces. The pieces were then incubated with Hank's balanced salt solution (HBSS)-EDTA for 10 min on a shaker at 37 **°**C, the supernatant containing epithelial cells was discarded and gut parts were washed twice with ice cold PBS. Afterwards the colon was digested once for 30 min and then twice for 20 min with a mixture of Collagenase IV (157 Wuensch units per ml, Worthington), DNAse I (0.2 mg ml^−1^ dissolved in PBS) and Liberase (0.65 Wuensch units per ml, both Roche, dissolved in Hank's Balanced Salt Solution with fetal calf serum (FCS)), the supernatant was collected after each digestion and the cells were washed once with PBS. Cells from all three digestions were combined and immune cells enriched using gradient centrifugation. For this cells were resuspended in 40% Percoll and this solution was overlayed onto a 80% Percoll solution. Centrifugation was carried out for 20 min at 1,800 r.p.m. and 4 **°**C without break. Cells at the interphase were collected, washed once and used for further analysis.

### Generation of bone marrow chimeras

To generate BM chimera recipient mice were irradiated with two split doses of 550 rad using a Cesium source (Gammacell 40, AECl, Mississauga, Canada). Irradiated animals were reconstituted with 5 × 10^6^ BM cells, 1:1 mixed from Ly5.1^+^ and Ly5.2^+^ BM. To prevent infection, animals received 1.2 g l^−1^ neomycin in water *ad libitum* for 4 weeks. Animals were analysed 8–10 weeks after reconstitution.

### Flow cytometry analysis

Where possible, 2 × 10^6^ cells were used for every staining with titred antibodies in PBS containing 2% FCS and 0.01% NaN_3_ (fluorescence-activated cell sorting (FACS) buffer) for 20 min at 4 °C in the dark. Cells were washed once and used for direct acquisition on BD FACSCanto or fixed using 2% paraformaldehyde in FACS buffer and measured the next day. Dead cells were always excluded using Aqua LIVE/DEAD Fixable Aqua DeadCell Stain Kit (Invitrogen, TermoFischer, Cat: L34957) or Zombie Aqua Fixable Viability Kit (BioLegend, Cat: 423102). For intracellular stainings cell were fixed and permeabilized after they have been stained for all extracellular markers. For the staining of FoxP3 cells were washed once and then resuspended in 200 μl 1 × Fixation/Permeabilization solution (eBioscience, Cat: 00-5523-00) for at least 30 min at 4 **°**C in the dark. Cells were spun down, the supernatant removed and the cells washed twice with 1 × permeabilization buffer (eBioscience, Cat: 00-5523-00). Cells were then stained with intracellular antibodies in 50 μl permeabilization buffer for 30 min at 4 **°**C in the dark. Afterwards cells were washed once and acquired by FACS. For intracellular cytokine stainings cells were fixed and permeabilized using BD Cytofix/Cytoperm (Fixation and Permeabilization Solution, BD Biosciences, Cat: 51-2090KZ) and BD Perm/Wash (Buffer, BD Biosciences, Cat: 51-2091KZ) according to manufacturers' instructions. Acquisition was either performed using a FACSCalibur or FACSCanto II. Cell sorting was performed at FACSAria (all BD). The following antibodies were used: 33D1 (33D1; FITC; dil. 1:400), CD3 (145-2C11; PE-Cy7, dil. 1:400), CD11b (M1/70; APC-eFluor780, dil. 1:400), CD11c (N418; PE-Cy7, dil. 1:600; APC, dil. 1:100), CD25 (PC61.5; PerCP-Cy5.5, dil. 1:400), CD70 (FR70; Biotin, dil. 1:400), CD80 (16-10A1; PE, dil. 1:400), CD205 (205yekta; APC, dil. 1:500), Esam (1G8; PE, dil. 1:100), F4/80 (BM8; PE-Cy7, dil. 1:400), FoxP3 (FJK-16s; eFlour660, dil. 1:50), Helios (22F6; FITC, dil. 1:400), MHCII (M5/114.15.2; FITC, dil. 1:800, PerCP-Cy5.5, dil. 1:800), RORgt (AFKJS-9; PE, dil. 1:400), IFN-γ (XMG1.2; FITC, dil. 1:500; APC, dil. 1:400), IL-17-A (TC11-18H10.1; PE, dil. 1:200) and Ly6C (AL-21; FITC, dil. 1:400) (eBioscience); CD86 (GL-1; PE, dil. 1:1,000), CD103 (M290; BV421, dil. 1:150; PE, dil. 1:150) (BD Pharmingen); CD4 (GK1.5; APC-Cy7, dil 1:800), CD8α (MCD0826; PE, dil. 1:400; APC-eFlour780, dil. 1:300; BV421, dil. 1:800) (Invitrogen); CD45 (30.F11; APC-eFlour780, dil. 1:200), CD45.1 (A20; PE, dil. 1:400), CD64 (X54-517.1; APC, dil. 1:200), CD90.1 (OX-7; FITC, 1:400)(BioLegend); cleaved Caspase 3 (D3E9; unlabelled, dil. 1:200) (Cell Signaling, Cat: 51-2091KE); goat anti-Rabbit (PE, dil. 1:100) (Life technologies). Data analysis was performed using FlowJo version 8 and 9 (TreeStar, Ashland, OR, USA). Analysis was performed using FlowJo (Treestar).

### Depletion of commensal bacteria

To deplete as many commensal bacteria as possible, animals were provided with a mixture of ampicilin sodium salt (1 g l^−1^), vancomycin hydrochloride (500 mg l^−1^), neomycin sulfate (1 g l^−1^) and metronidazole (1 g l^−1^) in the drinking water for at least 3 weeks^61^.

### Transcriptional analysis

Total RNA from sorted cells was isolated using TRIZOL and cDNA was generated using QuantiTect Reverse Transcription Kit (QIAGEN, Cat No: 205311). TaqMan PCR was performed using the Universal Probe Library Set mouse (Roche) according to manufacturer's instructions. Gene expression was normalized to Ubiquitin c expression. The following Primers were used: Ubiquitin c forward 5′-GACCAGCAGAGGCTGATCTT-3′, reverse 5′- CCTCTGAGGCGAAGGACTAA-3′, probe # 11; IL-6 forward 5′-GAAGGGCACTGCAGGATAGA-3′, reverse 5′-TCCCCAGAGTGTGGCAGT-3′, probe # 12, IL-23p19 forward 5′-ATAGCCCCATGGAGCAACTT-3′, reverse 5′- GCTGCCACTGCTGACTAGAA-3′, probe # 25; Aldh2a forward 5′-CATGGTATCCTCCGCAATG-3′, reverse 5′-GCGCATTTAAGGCATTGTAAC-3′, probe # 33; Itgav forward 5′-GGTGTGGATCGAGCTTCTT-3′, reserve 5′-CAAGGCCAGCATTTACAGTG-3′, probe # 21. Relative expression was calculated using the ΔΔCt method. For nanostring analysis CD11c^+^ cells were FACS sorted. Cells were washed once and then resuspended in buffer RLT (Quiagen) to disrupt the cells and unfold all proteins. This was immediately snap-frozen and then kept at −80 °C. The gene expression was analysed using the mouse immunology kit-24rxn GXA-MIM1-24 for the nanostring platform (NanoString Technologies, Seattle, WA, USA). Samples were processed according to manufacturer's protocol.

### CD40 injection

To evaluate the influence of a CD40 signal on DCs animals were injected with 200 μg of anti-CD40 mAb clone FJK45 intraperitoneally and animals were sacrificed by cervical dislocation at the indicated time points.

### ELISA for lipocalin

Faecal samples were reconstituted in PBS containing 0.1% Tween 20 (100 mg ml^−1^) and vortexed for 20 min for homogenisation. Upon centrifugation for 10 min at 12,000 r.p.m. supernatants were analysed for lipocalin-2 content using Quantikine ELISA kit for mouse Lipocalin-2/NGAL (R&D Systems, Cat: MLCN20).

### ELISA for commensal-specific antibodies

The cecum of C57BL/6 mice was removed, opened longitudinally, transferred into a 2 ml Eppendorf cup, containing 1.5 ml PBS and cecal content was expelled by vigorously vortexing. Remaining cecal tissue was removed and PBS and cecal content was transferred into tubes with Lysing Matrix E (MP Biomedicals, Cat: 116914050) and then homogenized using the FastPrep-24 Instrument (MP Biomedicals, Cat: 116004500) for 45 s at maximum speed. Samples were spun down and supernatant was collected, filtered and spun again at maximum speed. The protein concentration was determined and the cecal bacterial lysate was stored at −20 °C until used. Cecal bacterial lysate was diluted in carbonate buffer to a final concentration of 50 ng ml^−1^ and 100 μl of this was coated per well over night at 4 **°**C. Wells were washed five times with PBS 0.05% (v/v) Tween20. Afterwards unspecific binding was blocked using 200 μl PBS with 0.5% (v/v) MMP for 2 h at room temperature and wells were then again washed five times with PBS 0.05% (v/v) Tween20. Serum of mice was diluted either 1:300 or 1:600 and 100 μl of this was added to a well, incubated for two hours at room temperature and washed again for five times with PBS 0.05% (v/v) Tween20. For detection of isotype specific antibodies coupled to horseradish peroxidase were used at a dilution of 1:4,000 in blocking buffer for 2 h at room temperature. After another round of washing the ELISA was developed using 100 μl of 3,3′,5,5′-tetramethylbenzidin solution. The reaction was stopped by adding 50 μl 2N H_2_SO_4_. Optical density was measured at a wavelength of 450 nm with 630 nm as a reference wavelength.

### Faecal IgA flow cytometry

Faecal pellets from 8- to 15-week-old mice were homogenized by bead beating (FastPrep-24 Instrument, MP Biomedicals, Cat: 116004500) and stained with PE Anti-Mouse IgA (1.3 μg ml^−1^ eBioscience, clone mA-6E1).

### Histopathology

Histopathological examination was performed on tissue samples of sex-matched, age-matched mice. Tissue samples were fixed in 4% neutral buffered formaldehyde solution at room-temperature for ∼24 h and embedded in paraffin or in glycolmethacrylate and methylmethacrylate (GMA/MMA). Sections of 1.5 μm (GMA/MMA), respectively of 3.0 μm (paraffin) thickness, were stained with haematoxylin and eosin (HE), and with Giemsa. All sections were evaluated in a blinded fashion.

### 16S rRNA amplicon sequencing and taxonomic profiling

Analysis of the intestinal microbiota of mouse faecal samples was based on the recently developed dual-index strategy for sequencing on the MiSeq Illumina platform^62^. Briefly, genomic DNA was extracted from stool samples using a phenol-chloroform extraction technique with mechanical disruption^63^. Inserts were PCR-amplified in duplicate using multiplexed 8 forward × 12 reverse primers targeting the V3-V4 variable regions of the 16S rRNA gene^64^ and purified using the Agencourt AMPure XP PCR Purification system (Beckman Coulter, Krefeld, Germany, Cat: A63880). Purified amplicons were combined in equimolar amounts in one pool, and sent to Eurofins Genomics (Ebersberg, Germany) for library quality control and sequencing on the Illumina MiSeq v.3 as 300-bp paired-end runs. Sequencing output was pre-processed to retain only high-quality reads, which were then analysed with QIIME v 1.8 (ref. 65). Open-reference OTU clustering and taxonomy assignment of sequences were done with UCLUST^66^ against the Silva database Release 111 (ref. 67) at the 97% similarity level. Alpha diversity was calculated on rarefied OTU tables using the observed OTUs metric. 16S rRNA amplicon sequencing data have been deposited in the NCBI Sequence Read Archive under Accession Number SRX1799186.

### Statistics

For absolute cell numbers the percentage of living cells of a certain subset was multiplied by the number of living cells as determined by CASY Counter. If not mentioned otherwise, significance was determined using the Student's *t*-test and defined as follows: *P<0.05, **P<0.01 and ***P<0.001. Bar graphs show mean±s.e.m. for the group sizes as indicated in the figure legends.

### Data availability

Sequence data that support the findings of this study have been deposited in NCBI Sequence Read Archive with the primary accession code SRX1799186. The other data that support the findings of this study are available from the corresponding author upon request.

## Additional information

**How to cite this article:** Barthels, C. *et al*. CD40-signalling abrogates induction of RORγt^+^ Treg cells by intestinal CD103^+^ DCs and causes fatal colitis. *Nat. Commun.* **8,** 14715 doi: 10.1038/ncomms14715 (2017).

**Publisher's note:** Springer Nature remains neutral with regard to jurisdictional claims in published maps and institutional affiliations.

## Supplementary Material

Supplementary Information Supplementary Figures 1-7 and Supplementary Methods
