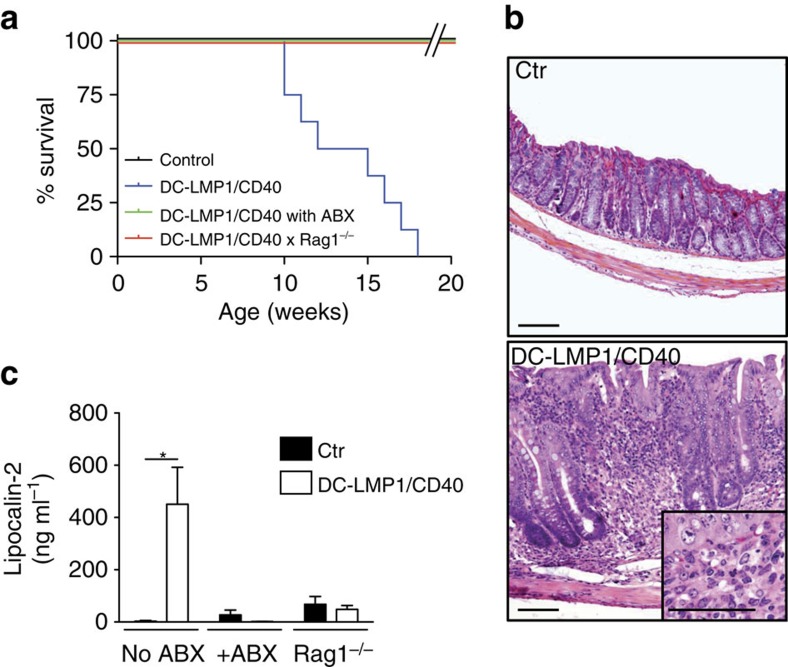# Erratum: CD40-signalling abrogates induction of RORγt^+^ Treg cells by intestinal CD103^+^ DCs and causes fatal colitis

**DOI:** 10.1038/ncomms15439

**Published:** 2017-04-24

**Authors:** Christian Barthels, Ana Ogrinc, Verena Steyer, Stefanie Meier, Ferdinand Simon, Maria Wimmer, Andreas Blutke, Tobias Straub, Ursula Zimber-Strobl, Esther Lutgens, Peggy Marconi, Caspar Ohnmacht, Debora Garzetti, Bärbel Stecher, Thomas Brocker

Nature Communications 8: Article number: 14715; DOI: 10.1038/ncomms14715 (2017); Published: 03 09 2017; Updated: 04 24 2017

In Fig. 2 of this Article, the inset image in the bottom panel was inadvertently omitted during the production process. The correct version of the Fig. 2 is shown below as Fig. 1. 

## Figures and Tables

**Figure 1:**