# Supplementary material for: CD40-signalling abrogates induction of RORγt+ Treg cells by intestinal CD103+ DCs and causes fatal colitis
Source: Nat Commun. 2017 Mar 9;8:14715. doi: 10.1038/ncomms14715 (PMC5347138; doi:10.1038/ncomms14715)
Supplement: Supplementary Information — Supplementary Figures 1-7 and Supplementary Methods [file ncomms14715-s1.pdf]

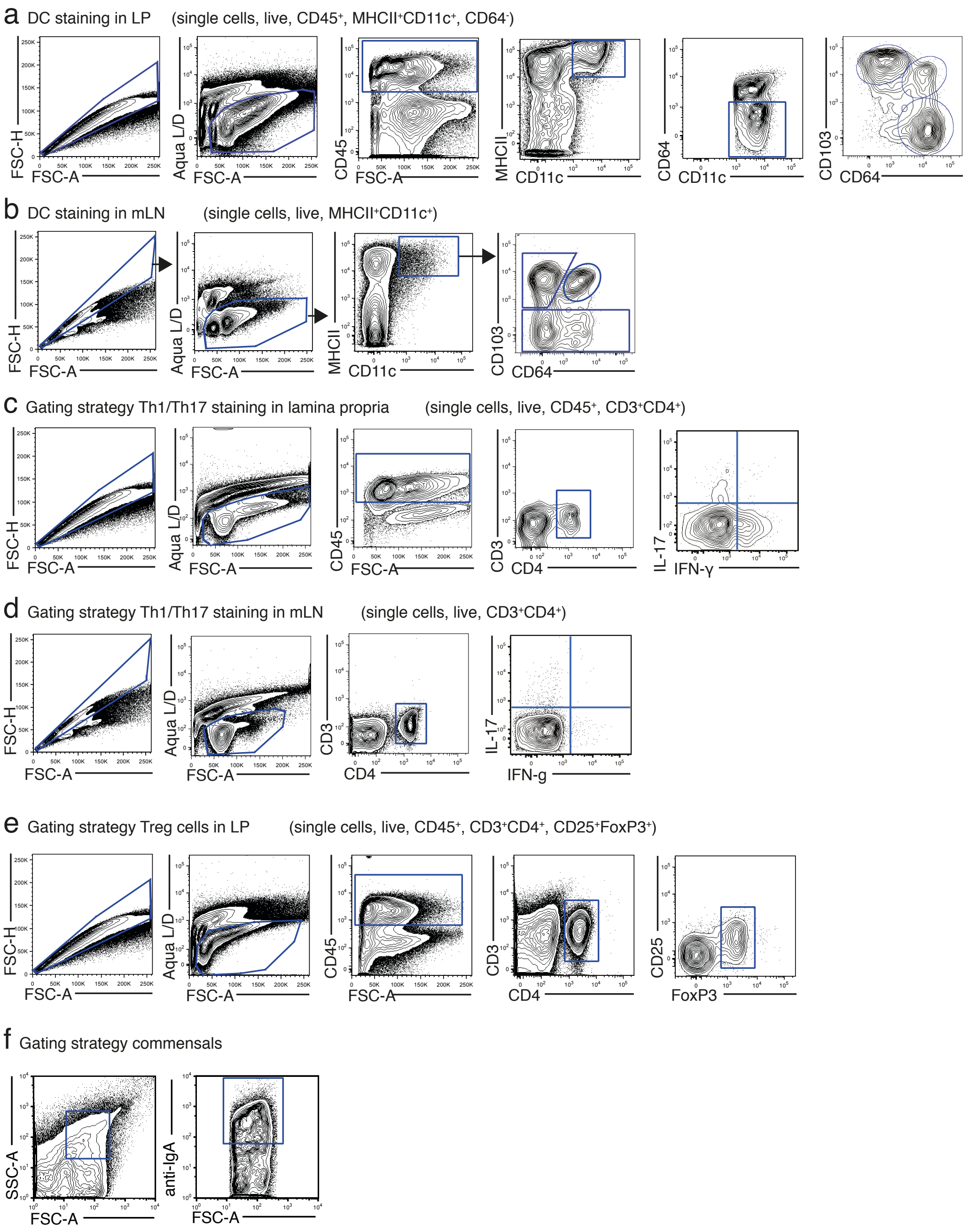

Supplementary Figure 1: Gating strategies for FACS analyses of different cells.

Gating strategies to differentiate the different DC subpopulations in a) the lamina propria and b) the mesenteric lymph nodes as applied in Fig. 1a, 1d, 3a, 3b, 3c, 3d. Gating strategies for analysis of Th1/Th17 cells in c) the lamina propria and d) the mesenteric lymph nodes as used in Fig. 5a. Il-17 and IFN-γ staining were performed intracellularly. e) Gating strategy for Fig. 4a, 4b, 4c for analysis of CD25$^+$Foxp3$^+$ Treg cells in the lamina propria. f) Analysis of commensals from fecal pellets of mice as shown in Fig. 5d.

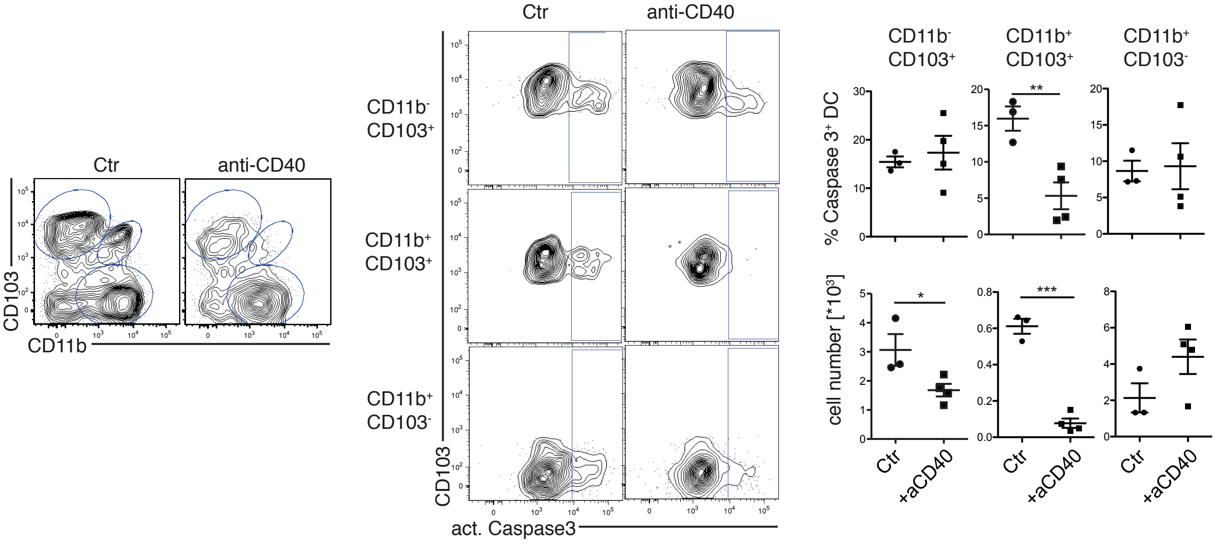

Supplementary Figure 2: Mice were injected with 200 µg anti-CD40 mAb and DC from LP were analysed 24 hours later. Dendritic cells (live, single cells, $CD45^{+}MHCII^{+}CD11c^{+}CD64^{-}$; gating not shown) were further analysed for subpopulations with CD103 and CD11b as shown in the left panel. Gates of the three main subpopulations are shown and were further analysed for active Caspase3 (middle panel). The percentages and total numbers of the active caspase 3^{+} DC are shown in the bar graphs (right-hand panel). One representative experiment out two is shown. Depicted is the mean ± s.e.m. of (Ctr, n=3, +anti-CD40, n=4) individual female 8-10 wk old animals per group. *P<0.05; **P<0.01; ***P<0.001; n.d., two-tailed unpaired t-test.

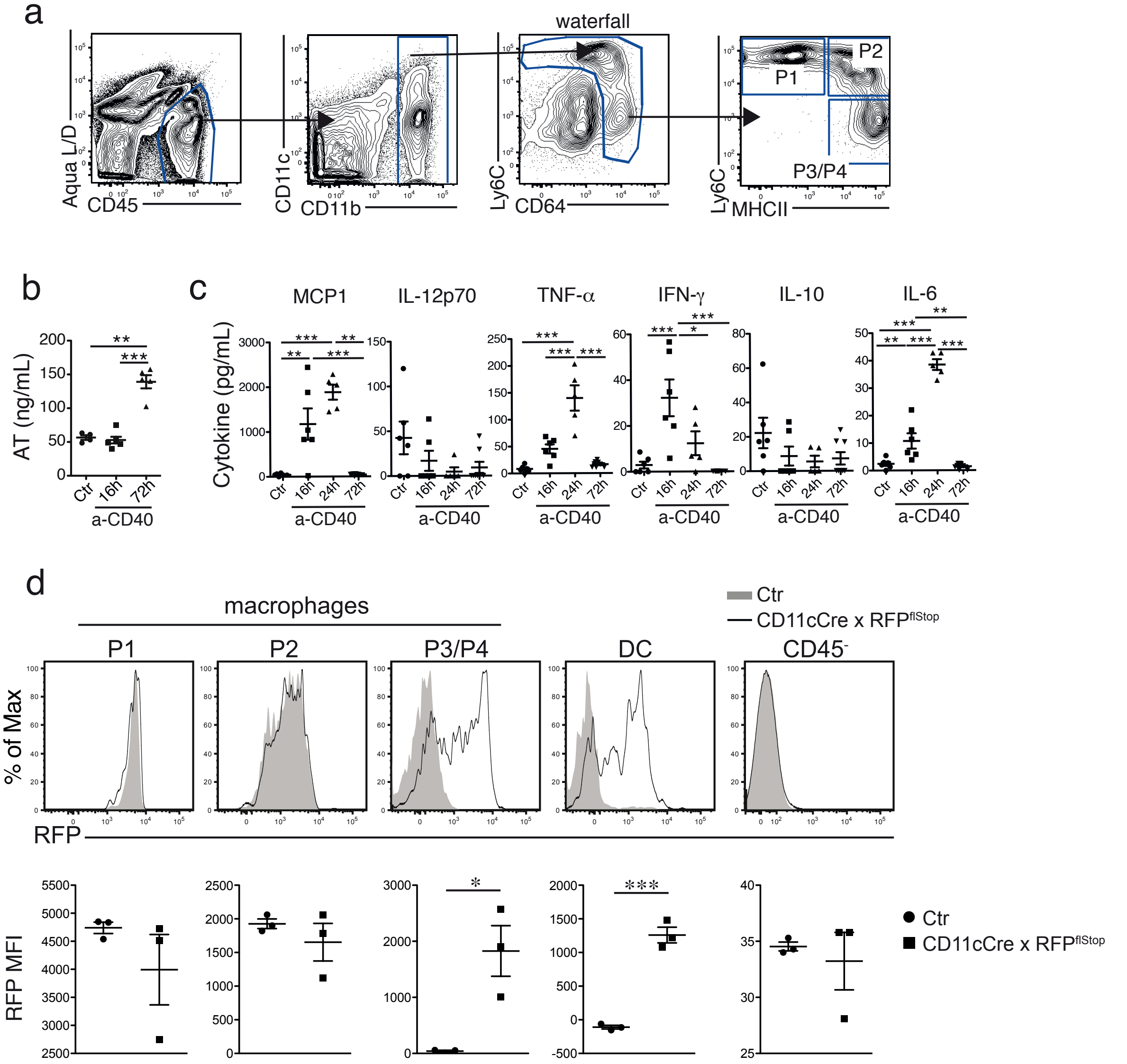

Supplementary Figure 3. Gating strategy for macrophages and serum analyses of CD40-injected mice

a) Gating strategy to differentiate the different stages of macrophage development in the lamina propria. **b**) Liver damage was assed by measuring the serum levels of the liver specific enzyme alanine-aminotransferase at the indicated time points after anti-CD40 injection via ELISA. **c**) Serum cytokine levels for the indicated cytokines were measured at different time points after anti-CD40 injection using a bead based flow cytometry assay. Data represent pooled results from two independent experiments. **d**) Expression of RFP in different cell subsets was analyzed in CD11cCre x RFP^{flstop} mice. Upper panel shows representative histograms, lower panel statistics. Dot blots and graphs show representatives of two independent experiments. Depicted is the mean ± s.e.m. of (*n*=3) individual 8 wk old female animals per group. *P<0.05; **P<0.01; ***P<0.001; two-tailed unpaired t-test.

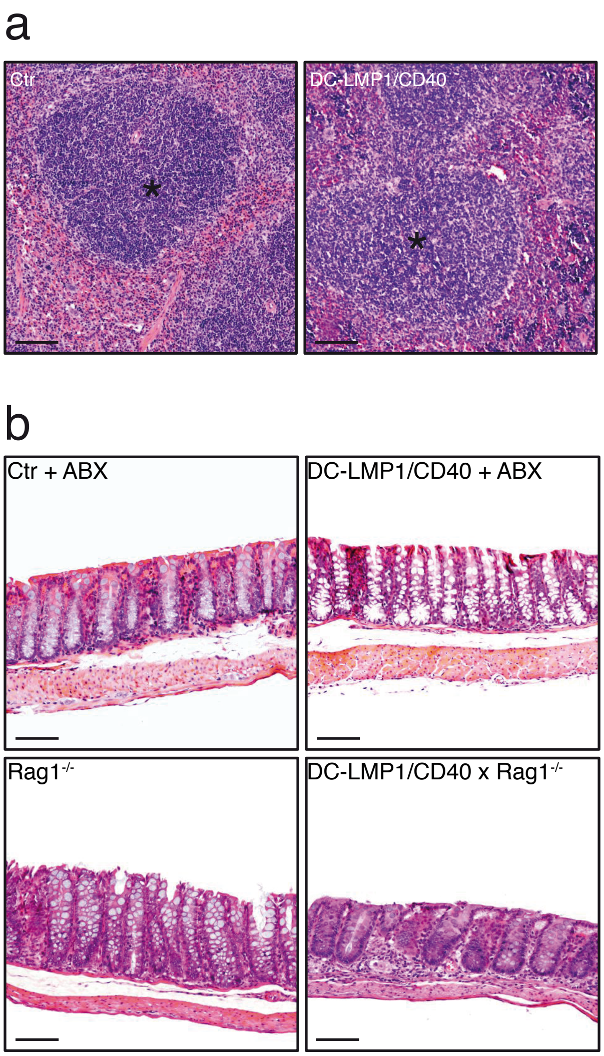

Supplementary Figure 4: DC-LMP1/CD40 animals do not show signs of colitis after ABX treatment or on the Rag1$^{-/-}$ background

a) The spleens of control (Ctr) and DC-LMP1/CD40 mice display normal microscopic white pulp (asterisk) morphology. Paraffin sections. HE-staining. Bars = 100 µm. **b**) Development of colitis is prevented in DC-LMP1/CD40 mice by ABX-treatment for 4 weeks (upper panel). DC-LMP1/CD40xRag1$^{-/-}$ mice show mild proprial mononuclear cell infiltration in the mucosa of the colon (lower panel) paraffin sections. HE-staining. Scale bars = 100 µm.

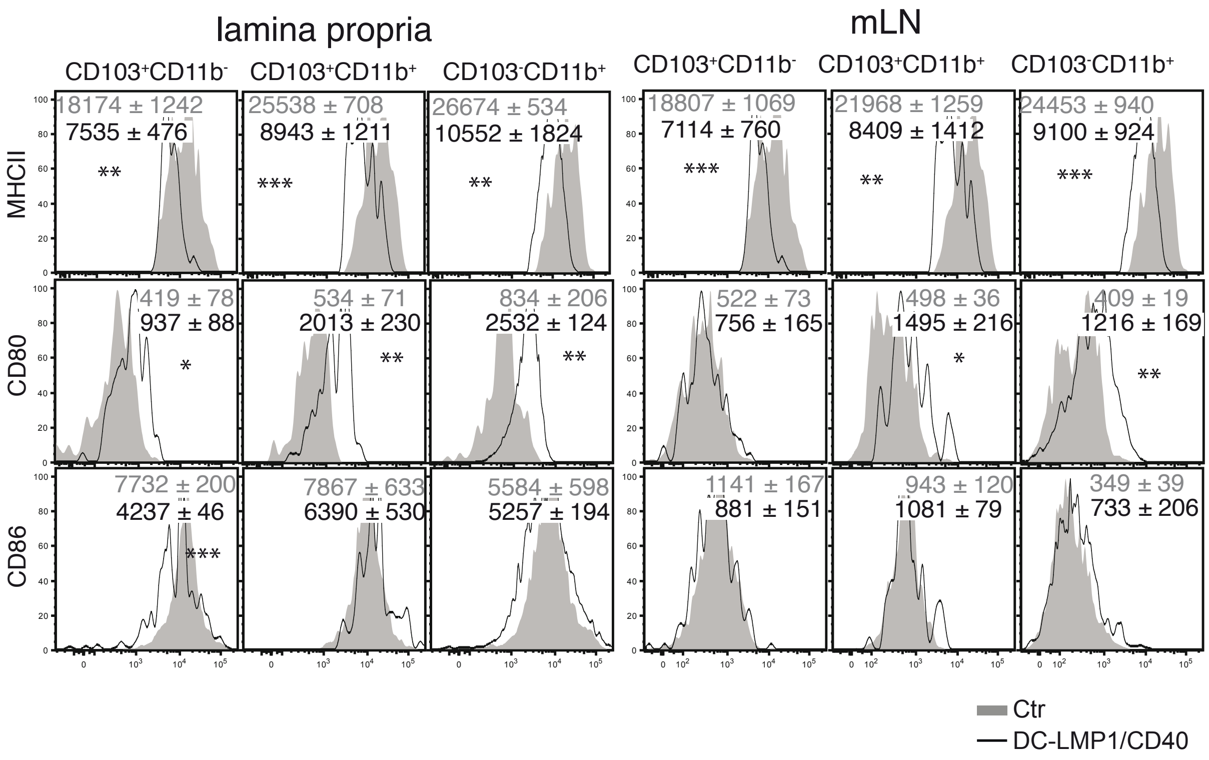

Supplementary Figure 5: Surface and gene expression analysis of DCs from DC-LMP/CD40-mice
DC subsets of different organs were analyzed for the expression of different activation markers. Shown are representative histograms of controls (grey) and DC-LMP1/CD40 (black line). Numbers represent mean fluorescence intensity. Dot blots and graphs show representatives of three independent experiments. Depicted is the mean ± s.e.m. of (*n*=3) individual 8-10 wk old female animals per group. *P<0.05; **P<0.01; ***P<0.001; two-tailed unpaired t-test.

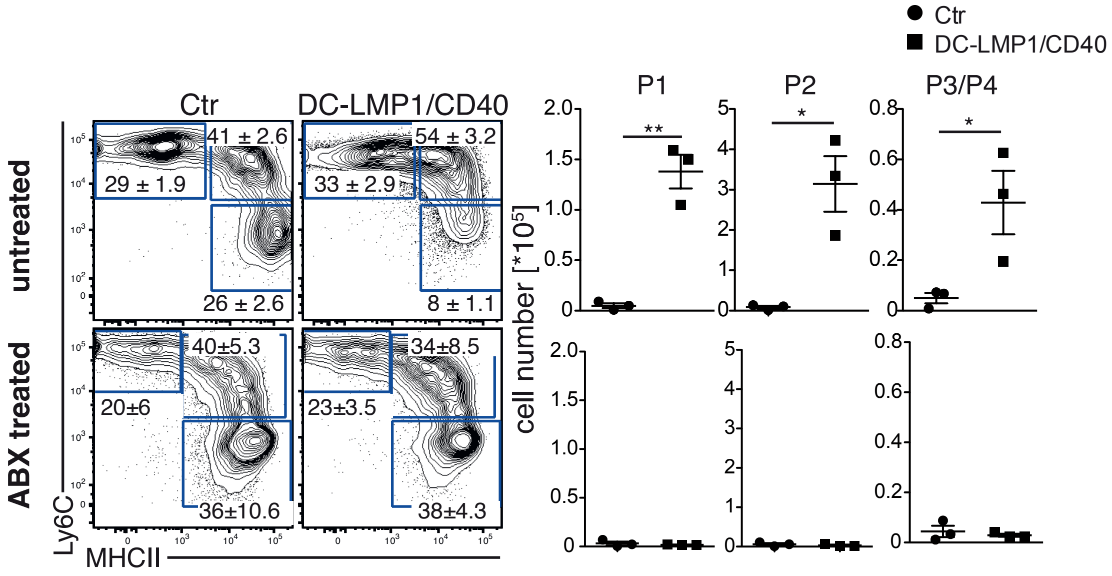

Supplementary Figure 6: Macrophages in the colon of DC-LMP/CD40 mice

Macrophages in the LP were analyzed using "waterfall" staining shown in supplemental figure 2a, in untreated and ABX-treated animals. Shown are representative FACS-plots, numbers indicate frequency of cells in each subset and bar graphs show absolute number per colon (n = 3). Dot blots and graphs show representatives of two independent experiments. Depicted is the mean ± s.e.m. of (n=3) individual 8-10 wk old female animals per group. *P<0.05; **P<0.01; two-tailed unpaired t-test.

chimeras:
Ctr+Ctr>Ctr = Ctr (CD45.1) + Ctr (CD45.2) > Ctr (CD45.1)
Ctr+Tg>Ctr = Ctr (CD45.1) + DC-LMP/CD40 (CD45.2) > Ctr (CD45.1)

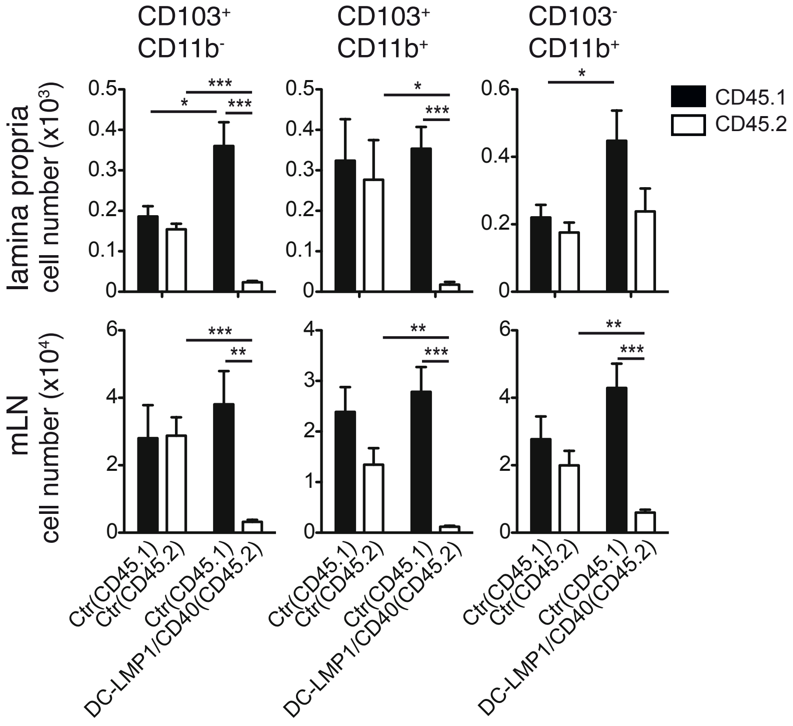

Supplementary Figure 7: Transgenic influence on CD103^{+}DC is cell intrinsic

To assess whether the LMP1/CD40-transgene effects only the DCs in which it is expressed, mixed bone marrow chimeras were prepared as shown at the top of the figure. Numbers of DCs within the respective subset are shown for the two different subsets of chimera. Dot blots and graphs show representatives of two independent experiments. Depicted is the mean ± s.e.m. of (n = 6) individual 8-10 wk old female animals per group. *P<0.05; **P<0.01; ***P<0.001; two-tailed unpaired t-test.
